# Music therapy for patients with depression: systematic review and meta-analysis of randomised controlled trials

**DOI:** 10.1192/bjo.2025.10822

**Published:** 2025-09-09

**Authors:** Youn Joo Lee, Seog Ju Kim, Jihyun Yoon, Jung Hwan Lee

**Affiliations:** Chadwick International School, Incheon, South Korea; Department of Psychiatry, Sungkyunkwan University School of Medicine, Samsung Medical Center, Seoul, South Korea; College of Music, The University of Suwon, Hwaseong, South Korea; Department of Rehabilitation Medicine, Namdarun Rehabilitation Clinic, Yongin City, South Korea

**Keywords:** Depression, mood disorder, music therapy, systematic review, meta-analysis

## Abstract

**Background:**

Depression is one of the most common mental diseases, leading to a decline in both psychiatric and physical functions. One non-pharmacological therapeutic strategy for the management of psychiatric disorders is music therapy.

**Aims:**

To assess the clinical effectiveness of music therapy and its various subscales for managing depressive symptoms (primary outcome) and related problems (secondary outcome) in comparison with other conventional treatments.

**Method:**

A comprehensive search of MEDLINE, Embase, Cochrane Review, CINAHL, PsyInfo and KMbase was conducted to identify randomised controlled trials published up to 31 August 2023. Studies assessing the clinical effectiveness of music therapy for individuals with depression were included, and data on participants, music therapy and clinical measurement scores were extracted. This study was registered with PROSPERO (no. CRD42023466833).

**Results:**

Music therapy was significantly more effective than controls in reducing depressive symptoms (standardised mean difference (SMD) −0.97 [95% CI: −1.23 to −0.71], *P* < 0.01). This benefit was consistent regardless of music therapy types, delivery methods or provider professionalism. In addition, music therapy was significantly better than controls in improving quality of life (SMD 0.51 [95% CI: 0.19−0.83], *P* < 0.01) and sleep quality (SMD −0.61 [95% CI: −1.03 to −0.19], *P* < 0.01), although it showed only a non-significant trend towards reducing anxiety (SMD −0.98 [95% CI: −2.01 to 0.06], *P* = 0.06). The evidence level was very low due to high risk of bias, inconsistency due to high heterogeneity and imprecision.

**Conclusions:**

Despite the very low evidence level, music therapy may be recommended with weak strength for patients with depression, considering the results of the meta-analysis and the high accessibility and broad applicability of music.

Depression is a mood disorder and among the common mental illnesses, affecting hundreds of millions of people worldwide. It can lead to various emotional and physical problems and reduce an individual’s ability to function at work and home.^1^ Depression is associated with a wide range of medical conditions, including neurological disorders such as dementia and Parkinson’s disease, stroke and musculoskeletal and cardiovascular diseases, as well as cancer.^2–4^ When co-occurring with other medical conditions, depression can discourage people from seeking treatment, leading to deterioration in health and worsening of depressive symptoms.^4^ Furthermore, depression impairs an individual’s ability to maintain relationships with family members, perform well at work and live a normal life. Ultimately, it contributes to a substantial economic and social burden by increasing healthcare costs and reducing life expectancy.^5^ Although psychotherapy and pharmacotherapy are the most common treatment options for depression, the results are not always satisfactory.^ 6^ Intractable depression persists over time and can lead to serious physical and psychological impairment, sometimes resulting in devastating outcomes such as suicide.^1^ In addition, emotional barriers to diagnosis, social stigma and the adverse effects associated with medication often cause patients to hesitate in seeking treatment, thereby worsening their condition.^7^ Therefore, therapeutic options that are more familiar to, or likely to be tolerated by, the individual are required. In this regard, music can serve as a favourable adjunctive therapeutic option because it can be tailored to patients’ preferences. Music therapy, defined as the professional use of music and its elements as an intervention, has been employed effectively to manage psychological and emotional issues caused by various medical, psychiatric and behavioural disorders.^8–12^

The American Music Therapy Association defines music therapy as the clinical and evidence-based use of music to accomplish individualised goals within a therapeutic relationship by a credentialled professional who has completed an approved music therapy programme.^1,13^ Music therapy is categorised into active music therapy, receptive music therapy and music medicine. In active music therapy, the subject engages in improvisation, songwriting, playing of musical instruments or singing with therapists or other participants. In receptive music therapy, individuals listen to music rather than creating it; however, this approach emphasises interaction with a therapist using various techniques such as providing music-related imagery, analysing lyrics and recalling feelings, memories or experiences associated with music. In music medicine, patients are simply instructed to listen to music. This approach focuses on the direct effects of music alone, independent of the patient–therapist relationship, which distinguishes it from receptive music therapy.^14^ All three subscales of music therapy have been found to enhancesubjects’ ability to cope with emotional distress.^15^ These effects are believed to be obtained by modulating the neuroendocrine system and activating the limbic system, which is associated with emotional status.^16–18^

It is both necessary and beneficial to evaluate the efficacy of music therapy and to determine whether its individual subscales, based on different categories and delivery methods, are effective in treating depressive symptoms and related clinical deficits in those with depression and other underlying diseases. This study aimed to evaluate the clinical effectiveness of music therapy and its various subscales in managing those with depressive symptoms (primary outcome) and associated problems (secondary outcome), in comparison with other conventional treatments.

## Method

### Data source

This systematic review was conducted following the Cochrane Collaboration guidelines for the systematic reviews of interventions. The review adhered to the Preferred Reporting Items for Systematic Reviews and Meta-analyses extension statement for reporting systematic reviews of healthcare interventions. The study protocol was registered in PROSPERO in October 2023 (no. CRD42023466833). A comprehensive search of the published literature was conducted in the databases Medline (PubMed), Embase, Cochrane Review, CINAHL, PsyInfo and KMBase for articles published up to 31 August 2023, using the individual search terms listed in the Supplementary material available at https://doi.org/10.1192/bjo.2025.10822.

### Study selection

The inclusion criteria included: randomised controlled trials (RCTs) published in either Korean or English; RCTs involving patients of any age with any underlying diseases and who were diagnosed with depression, using either a self-rated depression questionnaire or a validated clinician-rated assessment method; and RCTs evaluating any type of music therapy, including active music therapy, receptive music therapy and music medicine, delivered through either individual- or group-based therapy. Studies that did not clearly indicate depression diagnosed using a validated method at baseline were excluded. Among those studies that met the criteria above, only RCTs that reported clinical outcomes following music therapy in comparison with a control group were finally selected.

Article selection was based initially on title and abstract screening, followed by full-text review. The selection process for both title and abstract screening and full-text review was independently performed by two reviewers (Y.J.L. and J.H.L.), and any discrepancies were resolved by discussion with the entire research group (Y.J.L., S.J.K., J.Y. and J.H.L.).

### Data extraction and quality assessment

Data extraction was performed independently by three reviewers (Y.J.L., J.H.L. and J.Y.), and discrepancies were resolved through discussion or consultation with a fourth reviewer (S.J.K.). Data were extracted from the selected studies, including the characteristics of the participants (age, gender and diagnosis); music therapy subscales (music therapy types – active music therapy, receptive music therapy and music medicine); music therapy delivery method (group- or individual-based ); staff profession (certified music therapist or non-music therapist); validated clinical measurement scores of depression; and other associated clinical problems, such as quality of life (QOL), anxiety and sleep quality. These data were considered clinically meaningful and commonly associated with depression, and were sufficiently reported across the selected studies to allow for meta-analysis. Dichotomous variables, such as the number of participants, were extracted to estimate the relative risk ratio. Continuous variables, such as the mean and standard deviation of clinical scores, were extracted to estimate mean differences. If standard deviations were not reported, these were calculated from the confidence interval, mean and number of patients. Data extraction began on 15 October 2023.

Quality assessment of each study and evidence level were conducted using the methodology Grading of Recommendations Assessment, Development and Evaluation (GRADE).^19–21^ The bias assessment for each RCT was conducted following the method Risk of Bias (ROB), which consisted of seven domains: random sequence generation, allocation sequence concealment, blinding of participants and personnel, blinding of outcome assessment, incomplete outcome data, selective reporting and other biases.^22^ Each of the domains was rated as either ‘low risk’, ‘high risk’ or ‘unclear’. These evaluations were independently performed by two reviewers (Y.J.L. and J.H.L.), and disagreements were resolved through discussion with the entire research group.

Based on a comprehensive evaluation of inconsistency, indirectness, imprecision and publication bias, along with ROB assessment, the evidence level was graded as either high, moderate, low or very low. Furthermore, the recommendation strength was determined as either strong or weak following a comprehensive assessment of not only the evidence level, but also other factors such as benefits and risks, cost-effectiveness and applicability or accessibility. Both evidence level and recommendation strength were determined through discussion with the entire research group.

### Data synthesis and analysis

Meta-analysis was conducted using Review Manager software (RevMan version 5.4, The Cochrane Collaboration 2014) to compare clinical outcomes, including depression, QOL, anxiety and sleep quality scores, between the music therapy and control groups within 3 months following treatment. The main comparisons were as follows: (a) music therapy versus control (usual care, such as psychotherapy or pharmacotherapy) for alleviation of depression (1-1) subgroup analysis for active control (such as psychotherapy and pharmacotherapy) and passive control (rest and waiting) (1-2) subgroup analysis, based on whether aetiology or underlying disease was identified; (b) music therapy versus control for improvement of QOL; (c) music therapy versus control for amelioration of anxiety symptoms; and (d) music therapy versus control for improvement of sleep quality. To elucidate the clinical effectiveness of music therapy in subscales, subgroup analysis was conducted according to the following comparisons: (a) active music therapy versus control; (b) receptive music therapy versus control; (c) music medicine versus control; (d) group-based music therapy versus control; (e) individual-based music therapy versus control; (f) music therapist versus control; (g) non-music therapist versus control; and (h) music therapy versus other art therapies such as painting, poetry and creative play. Insufficient parameters, such as pain score, activities of daily living and adverse response scale, were not evaluated in the meta-analysis.

The results are expressed as mean difference and 95% CI for continuous outcome data, or as relative risk ratio and 95% CI for dichotomous outcome data. Pooled effects were estimated using standardised mean difference (SMD) and 95% CI, due to the use of different depression measurement scales across the selected studies. A *P*-value of <0.05 was considered statistically significant.

A random-effects model was used to estimate effect sizes and their statistical significance, based on the assumption that the participants and methods of the included studies, conducted by independent researchers, were not equivalent and therefore could not share a common effect size. Heterogeneity was assessed using *I* ^2^ statistics, with a value of *P* < 0.05 being considered as indicating a significantly high degree of heterogeneity.

## Results

### Characteristics of RCTs

**Fig. 1:**
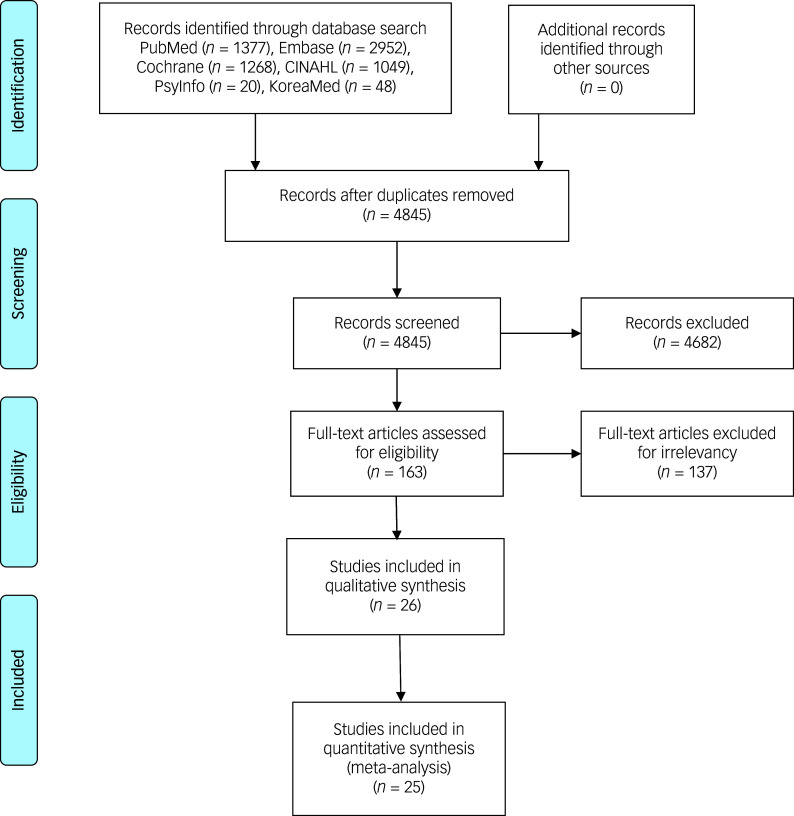
Flow diagram of study selection.

Initially, 6714 articles were identified from the database search. Following the removal of 1869 duplicates, 4845 potentially eligible articles remained. Title and abstract screening further led to the exclusion of 4682 articles that did not meet the inclusion criteria. The remaining 163 articles were included in the full-text analysis, of which 137 were subsequently excluded due to irrelevance to the scope of this analysis. Consequently, a total of 26 RCTs were included in this study (Fig. 1 and Tables 1–3). Of these, 25 articles containing continuous data were included in quantitative synthesis (meta-analysis), excluding one article^23^ that provided only dichotomous data.

**Table 1:** Statistics derived from the 26 studies: demograhic data

Author(s), nation	Diagnostic criteria	Comorbid diseases	Number of patients (% female)	Age (years): mean (s.d.) or age group, number
Albornoz^41^ Venezuela	Beck Depression Inventory ≥10	Substance abuse	MT 12 (0) CG: 12 (0)	The age of each group was not identified.
An et al^30^ China	Hamilton Depression Scale-24: 8–35	Cancer	MT: 60 (41.7) CG: 60 (38.3)	MT: 55.76 (8.81) CG: 56.05 (8.73)
Arruda et al^29^ Brazil	Beck Depression Inventory ≥5	Cancer pain	MT: 22 (72.7) Poetry CG: 22(72.7) Standard CG: 21(71.4)	18–40/41–60/>60 years MT: 8/7/7 Poetry CG: 2/10/10 Standard CG: 4/10/7
Atiwannapat^24^ Thailand	ICD-10 Montgomery–Åsberg Depression Rating Scale ≥7	Cancer	Active: 5 (80) Receptive: 5 (60) CG: 4(100)	Active: 41.6 (11.15) Receptive: 54.4 (6.73) CG: 55.25 (10.21)
Baker^25^ Australia	Montgomery–Åsberg Depression Rating Scale ≥8	Dementia	GMT: 77 (31.2) RCS: 82 (28) GMT + RCS: 79 (34.2) CG: 80 (31.2)	GMT: 86.0 (7.5) RCS: 87.1 (7.0) GMT + RCS CG: 85.8 (7.9) CG: 87.2 (6.5)
Carr et al^ 6 ^ UK	ICD-10		MT: 20 (65) CG: 10 (30)	MT: 42.25 (11.8) CG: 48.8 (10.85)
Castillo-Pérez et al^23^ Mexico	Diagnosis and initial patient selection was based on Zung Self-rated Depression scale, but score for diagnosis was not identified.		MT: 41 (80.5) CG: 38 (84.2)	25–35/36–45/46–60 years MT: 14/15/12 CG : 12/10/16
Chan et al^16^ Singapore	Diagnosis and initial patient selection was based on Geriatric Depression Scale, but score for diagnosis was not identified.		MT: 23(56.5) CG: 23(54.2)	60–64/65–69/70–74/75–79/80 years MT: 5/4/4/5/5 CG: 4/4/5/6/5
Chan et al^34^ Singapore	Geriatric Depression Score-15 ≥3		MT: 21 (57.1) CG: 21 (52.4)	60–64/65–69/70–74/75–79/80 years MT: 2/2/3/5/9 CG: 0/6/5/4/6
Chan et al^ 42 ^ Singapore	Geriatric Depression Score-15 ≥4		MT: 24 (62.5) CG: 26 (65.4)	55–64/65–74/75 years MT: 16/6/2 CG: 16/6/4
Chen et al^43^ Taiwan	Depression Mood Self-Report Inventory for Adolescence Score ≥8		MT: 31 (96.8) CG: 12(97.5)	MT: 18.5 (0.35) CG: 18.6 (0.48)
Daengruan et al^31^ Thailand	Patient Health Questionnaire Depression Screening ≥7		MT: 9 (77.8) CG: 12 (88.9)	MT: 29.4 (13.8) CG: 38.3 (17.9)
de Araujo Oliveira et al^36^ Brazil	Diagnosis and initial patient selection was based on Beck Depression Inventory, but score for diagnosis was not identified.	Parkinson’s disease	MT: 10 (40) CG: 9 (33.3)	MT: 64 (10) CG: 67 (6)
Deshmukh et al^35^ India	DSM-IV		MT: 25 CG: 25 Gender ratio of each group was not identified.	The age of each group was not identified.
Erkkilä et al^ 32 ^ Finland	ICD-10		MT: 33 (75.8) CG: 46 (80.4)	MT: 35.8 (9.0) CG: 35.5 (10.5)
Fancourt and Perkins^26^ UK	Edinburgh Postnatal Depression Scale ≥10	Postnatal depression	MT: 48 (100) Play CG: 42 (100) Standard CG: 44 (100)	MT: 35.3 (4.0) Play CG: 35.9 (4.2) Standard CG: 34.6 (3.4)
Gök Ugur et al^44^ Turkey	Geriatric Depression Scale ≥5		MT: 32 (37.5) CG: 32 (31.3)	MT: 75 (8.19) CG: 77.72 (7.57)
Hamid^37^ Iran	Diagnosis and initial patient selection was based on Beck Depression Inventory, but score for diagnosis was not identified.		MT: 15 (100) CG: 15 (100)	The age of each group was not identified.
Jain et al^15^ India	ICD-10		MT: 30 (60) CG: 30 (50)	21–30/31–40/41–50/51–60 years MT: 6/15/6/3 CG: 12/15/3/1
Lin et al^27^ China	Hamilton Depression Scale >7	Stroke	MT: 30 (43.3) Pharmacotherapy CG: 32 (46.9) Acupuncture CG: 30 (66.7)	MT: 68.8 (11.5) Pharmacotherapy CG: 69.7 (10.4) Acupuncture CG: 72.9 (10.4)
Park et al^38^ Republic of Korea	Children’s Depression Inventory ≥22	ADHD	MT: 30 (44.4) CG: 30 (27.8)	MT: male 11.76 (2.34) female 12.53 (2.52) CG: male 11.91 (2.77) female 12.43 (2.99)
Perkins et al^39^ UK	Edinburgh Postnatal Depression Scale ≥10	Postnatal depression	MT: 44 (100) CG: 45 (100)	Age >18 years The age of each group was not identified.
Rezazadeh et al^28^ Iran	Diagnosis and initial patient selection was based on Maria Kovacs Children’s Depression Inventory, but score for diagnosis was not identified.	Burns	MT: 20 (55) Painting CG: 20 (40) Standard CG: 20 (45)	MT: 10.1 (1.4) Painting CG: 9.9 (1.4) Standard CG: 9.6 (1.2)
Verrusio et al^33^ Italy	DSM-IV		MT: 12 (41.7) CG: 12 (66.7)	MT: 74.8 (8.0) CG: 76.1 (7.1)
Yu et al^45^ Taiwan	Diagnosis and initial patient selection was based on Geriatric Depression Scale – Short Form, but score for diagnosis was not identified.		MT: 32 (68.8) CG: 31 (41.9)	MT: 80.30 (6.88) CG: 79.03 (7.71)
Zhang et al^40^ China	Difficulties in Emotion Regulation Scale ≥101, Beck Depression Inventory ≥10 and depressive symptoms		MT: 36 (55.6) CG: 35 (48.6)	MT: 18.26 (0.56) CG: 18.27 (0.45)

MT, music therapy group; CG, control group; ICD-10, International Statistical Classification of Diseases and Related Health Problems; GMT, group music therapy; RCS, recreational choir singing; DSM-IV, Diagnostic and Statistical Manual of Mental Disorders-IV; ADHD, attention-deficit hyperactivity disorder.

**Table 2:** Statistics derived from the 26 studies: treatment methods

Author, nation	MT types/professionality of therapist/delivery method	Description of MT	CG treatment
Albornoz^41^ Venezuela	Active/non-professional/group	Improvisational MT, 2 h once per week for 3 months	Standard care
An et al^30^ China	Music medicine/non-professional/ individual	Chinese traditional music	Pharmacotherapy
Arruda et al^29^ Brazil	Music medicine/non-professional/individual		Poetry group, standard care
Atiwannapat^24^ Thailand	Active and receptive/professional/group	Active (a) instrument choir playing, (b) song writing and group performance and (c) improvisation on percussion; receptive (a) lyric analysis, (b) song writing and (c) drawing while listening to music; 1-h session once per week for 12 sessions	Group counselling
Baker^25^ Australia	Active/professional/group	GMT: familiar song singing, improvising on percussion, movement to music; RCS: song singing with lyrics on screen, 45 min twice per week for 3 months and thereafter once per week for 3 months (39 sessions over 26 weeks); GMT + RCS: twice per week each for 3 months and once per week for each intervention for 3 months (78 sessions over 26 weeks)	Standard care
Carr et al^ 6 ^ UK	Active/professional/group	90 min three times per week over 14 weeks	Wait-list control
Castillo-Pérez et al^23^ Mexico	Music medicine/professional/individual	Baroque and classical music; 50 min once per day at home and then once per week at the hospital for 8 weeks	Psychotherapy
Chan et al^16^ Singapore	Music medicine/non-professional/individual	Western classical and jazz, Chinese and Asian classical; 30 min once per week for 4 weeks	Uninterrupted rest
Chan et al^34^ Singapore	Music medicine/non-professional/individual	Chinese classical, Western classical and Western modern jazz; 30 min once per week for 4 weeks	Uninterrupted rest
Chan et al^ 42 ^ Singapore	Music medicine/non-professional/individual	Chinese, Malay, Indian and Western slow rhythmic music; 30 min once per week for 8 weeks	Uninterrupted rest
Chen et al^43^ Taiwan	Music medicine/non-professional/individual	Chinese five-element music therapy twice per week for 10 weeks	Routine lifestyle
Daengruan et al^31^ Thailand	Music medicine/non-professional/individual	At least three times per week	Noise muffling
de Araujo Oliveira et al^36^ Brazil	Music medicine/non-professional/individual		Physical therapy
Deshmukh et al^35^ India	Music medicine/non-professional/individual	45-min flute-based compositions based on raagas bahar, bihag, mishra pilu and malaymarutam; 1 h before bedtime for 4 weeks	Pharmacotherapy
Erkkilä et al^ 32 ^ Finland	Active/professional/individual	60 min, biweekly, total 20 sessions	Standard care
Fancourt and Perkins^26^ UK	Active/professional/group	60 min, once per week for 10 weeks	Creative play, standard care
Gök Ugur et al^44^ Turkey	Receptive/professional/individual	Turkish music, 3 days per week for 8 weeks	Not specifically mentioned
Hamid^37^ Iran	Active/non-professional/individual	Total 12 sessions	No treatment
Jain et al^15^ India	Receptive/non-professional/unidentified		Standard care
Lin et al^27^ China	Music medicine/non-professional/unidentified	Five phases of traditional Chinese music; music was selected based on the principle of treating emotional disturbance with hyperaffectivity	Pharmacotherapy, acupuncture
Park et al^38^ Republic of Korea	Active/professional/unidentified	50 min, twice per week for 3 months	Pharmacotherapy
Perkins et al^39^ UK	Active/professional/group	Weekly sessions for 6 weeks; free songwriting composition through discussion of ideas, refinement of lyrics and creation of melody, and group singing of songs	Standard care
Rezazadeh et al^28^ Iran	Receptive/professional/individual	After listening to instrumental music for 15 min, the researcher spoke with the child for about 30 min regarding his feelings about the music	Painting, standard care
Verrusio et al^33^ Italy	Music medicine/non-professional/individual	Listening to music with physical exercise for 1 h per week	Pharmacotherapy
Yu et al^45^ Taiwan	Receptive/non-professional/group	30 min twice per week for 10 weeks; music background and instrument introduction for 5 min; listening to live music performances for 15 min and sharing listening experiences for 10 min	Standard care
Zhang et al^40^ China	Active/professional/group	Freely playing percussion instruments and creating personalised music belonging to both individuals and the team; during the performance, the therapists follow the students’ performance synchronously to stimulate and amplify the students’ emotion	Not specifically mentioned

MT, music therapy group; CG, control group; GMT, group music therapy; RCS, recreational choir singing.

**Table 3:** Statistics derived from the 26 studies: evaluation methods and results

Author, nation	Evaluation tool for depression	Other clinical evaluation tools	Results
Albornoz^41^ Venezuela	Beck Depression Inventory, Hamilton Rating Scale for Depression		MT showed significantly better outcome in controlling depression.
An et al^30^ China	Hamilton Depression-24	Chinese version of the Tumour Quality of Life Scale	MT showed significantly better outcome in controlling depression and improving quality of life.
Arruda et al^29^ Brazil	Beck Depression Inventory	Visual Analogue Scale, Herth Hope Scale	MT and control group showed improvement in depression and hope score, but there was no statistically significant difference between the two groups.
Atiwannapat^24^ Thailand	Montgomery–Åsberg Depression Rating Scale	Thai Depression Inventory, Thai version of the Short Form Health-related Quality of Life Survey	Both active and receptive MT showed reduction of depression and improvement of QOL, but without statistical significance in comparison with control.
Baker^25^ Australia	Montgomery–Åsberg Depression Rating Scale	Neuropsychiatric Inventory Questionnaire, Quality of Life in Alzheimer’s Disease, Euro Quality of Life Five-Dimension, Professional Care Team Burden Scale	RCS achieved significant clinical benefits but GMT did not.
Carr et al^ 6 ^ UK	Montgomery–Åsberg Depression Rating Scale, Beck Depression Inventory II	Brief Symptom Inventory, Rosenberg Self-esteem Scale, General Perceived Self-efficacy Scale, Client Satisfaction Questionnaire, Work and Social Adjustment Scale, Manchester Short Quality of Life Scale, Life Skills Profile, Level of Hospitalisation, Client Services Receipt Inventory	MT showed improvement in clinical scales at 3 and 6 months in comparison with post-treatment, but there was no degree of statistical significance.
Castillo-Pérez et al^23^ Mexico	Beck Depression Inventory, Hamilton Depression Scale		MT showed greater improvement in depression score than control.
Chan et al^16^ Singapore	Geriatric Depression Scale		MT showed greater improvement in depression score than control.
Chan et al^34^ Singapore	Geriatric Depression Score-15	Pittsburgh Sleep Quality Index	MT showed greater improvement in depression score than control over the 4 weeks.
Chan et al^ 42 ^ Singapore	Geriatric Depression Scale		MT showed greater improvement in depression score than control at 4 weeks pos- treatment.
Chen et al^43^ Taiwan	Depression Mood Self-Report Inventory for Adolescence Scores		MT showed greater improvement in depression score than control.
Daengruan et al^31^ Thailand	Patient Health Questionnaire Depression Screening	Euro Quality of Life Five-Dimension, Medication Adherence Rating Scale	MT revealed a trend towards more favourable outcomes in clinical scales than control, but this was not statistical significant.
de Araujo Oliveira et al^36^ Brazil	Beck Depression Inventory		MT showed significantly greater improvement in depression score than control.
Deshmukh et al^35^ India	Montgomery–Asberg Depression Rating Scale	Pittsburgh Sleep Quality Index	Both MT and control showed comparable improvement in depression and sleep quality scores.
Erkkilä et al^ 32 ^ Finland	Montgomery–Åsberg Depression Rating Scale	Hospital Anxiety and Depression Scale-Anxiety Score, Global Assessment of Functioning, Health-related quality of life survey RAND-36, Toronto Alexithymia Scale	MT achieved better results in improvement of depression, anxiety and general functioning score.
Fancourt and Perkins^26^ UK	Edinburgh Postnatal Depression Scale		MT showed more significant and rapid improvement than control in individuals with moderate to severe symptoms.
Gök Ugur et al^44^ Turkey	Geriatric Depression Scale		MT showed lower depression scores than control.
Hamid^37^ Iran	Beck Depression Inventory	Oxford Happiness Questionnaire	MT achieved significantly better outcome in depression and happiness scores.
Jain et al^15^ India	21-item Depression Anxiety and Stress Scale		MT achieved better results in improvement of depression, anxiety and stress score.
Linet al^27^ China	Hamilton Depression Scale	Activities of Daily Living Scale, Treatment Emergent Signs and Symptoms Adverse Response Scale	MT and acupuncture showed significantly greater reduction in depression score than acupuncture only.
Park et al^38^ Republic of Korea	Children’s Depression Inventory	Daily Hassles Questionnaire	MT showed better clinical outcomes in depression and stress score than control.
Perkins et al^39^ UK	Edinburgh Postnatal Depression Scale	UCLA 3-Item Loneliness Scale, Social Connectedness Revised 15-item Scale	MT showed significantly better clinical outcomes in depression, loneliness and social connectedness scores.
Rezazadeh et al^28^ Iran	Maria Kovacs Children’s Depression Inventory	Spence Children’s Anxiety Scale, Five-Point Likert Scale	MT and painting resulted in significantly reduced depression and anxiety scores compared with the control group.
Verrusio et al^33^ Italy	Geriatric Depression Scale	Hamilton Anxiety Scale	MT achieved significantly reduced depression and anxiety scores at 3 and 6 months, but control achieved significant reduction in only anxiety score at 6 months.
Yu et al^45^ Taiwan	Geriatric Depression Scale – Short Form		MT showed significantly greater reduction of depression score than control at 5 and 10 weeks.
Zhang et al^40^ China	Beck Depression Inventory	Difficulties in Emotion Regulation Scale	MT succeeded in achieving significant reduction in depression and anxiety scores, but control failed to do so.

MT, music therapy group; GMT, group music therapy; RCS, recreational choir singing; QOL, quality of life.

In the meta-analysis, Atiwannapat et al’s study was divided into active and receptive music therapy groups, because it established two music therapy groups and compared these with the control group, respectively.^24^ Baker et al’s study was divided into three music therapy groups, including group music therapy (the GMT group), recreational choir singing (the RCS group) and group music therapy plus recreational choir singing (the GMT + RCS group), because each was compared separately with a control group.^25^ In addition, in the studies by Fancourt and Perkins, Lin et al, Rezazadeh et al and Arruda et al, two different control groups were used and analysed separately, because these studies included additional control groups such as poetry, creative play control, painting and acupuncture.^26–29^ Ultimately, 32 comparisons extracted from 25 studies were included in the meta-analysis.

The publication dates of the studies spanned from 2014 to 2023, with their sample sizes ranging from 14 to 318 participants. The studies were conducted across diverse regions, including Asia, Europe, Oceania and Middle and South America. In some Asian studies, traditional music specific to the country was used as a therapeutic method. All included trials evaluated depression using a validated assessment method. Each study compared the clinical effectiveness of music therapy for depression with usual care, pharmacotherapy, psychotherapy, rest or waiting. In addition, some studies examined improvements in other associated conditions such as anxiety, sleep quality, quality of life and self-esteem, alongside depressive symptoms.^ 6,24,25,28,30–35^ The characteristics of participants and music therapy, evaluation tools and clinical outcomes are listed in Table 1.

### Risk of bias

**Fig. 2:**
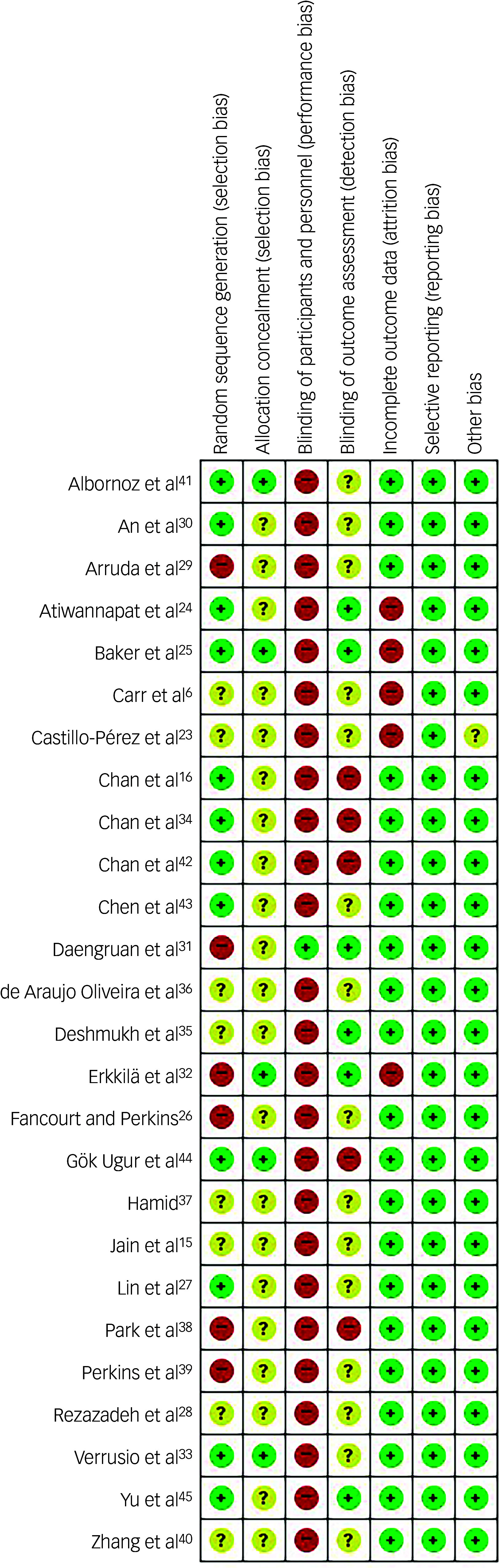
Quality assessment for extracted studies: risk of bias for randomised controlled study. Green, low risk of bias; red, high risk; yellow, unclear risk.

The ROB of all the selected studies is presented in Fig. 2. Of the 26 RCTs, 14 were assessed as having a high or unclear risk in the random sequence generation because the sequence generation process was not described.^ 6,15,23,26,28,29,31,32,35–40^ The most frequently biased domain was the blinding of participants and personnel (performance bias), with 25 studies considered high risk because patients or staff were aware of group allocation during treatment sessions, whereas only one study maintained blindness of patients and personnel during data collection and intervention, by providing noise-muffling on headphones rather than music in the control group while maintaining other conditions the same as for the music therapy group.^31^ A total of 20 studies were rated as high or unclear risk in the domain of blinding of outcome assessment (detection bias), due to lack of clarity or absence of assessor blinding.^ 6,15,16,23,26–30,33,34,36–43^ Overall, 97 of 182 domains (53.3%) were rated low risk and, therefore, the overall ROB was considered high (Fig. 2). Discrepancies between reviewers were initially observed in 26 of the total 182 domains (14.2%), all of which were resolved through discussion.

### Qualitative analysis

Among the 26 studies included in the final analysis, 21 reported that the music therapy group achieved a significant reduction in depression score compared with the control group, whereas the 5 other studies demonstrated that the music therapy and control groups had a comparable improvement in depression score.^ 6,24,29,31,35^ Notably, music therapy was superior to other treatments commonly used, including pharmacotherapy and psychotherapy, in managing patients with depression from various aetiologies (Tables 1–3).

### Quantitative analysis

#### Music therapy for depression

**Fig. 3:**
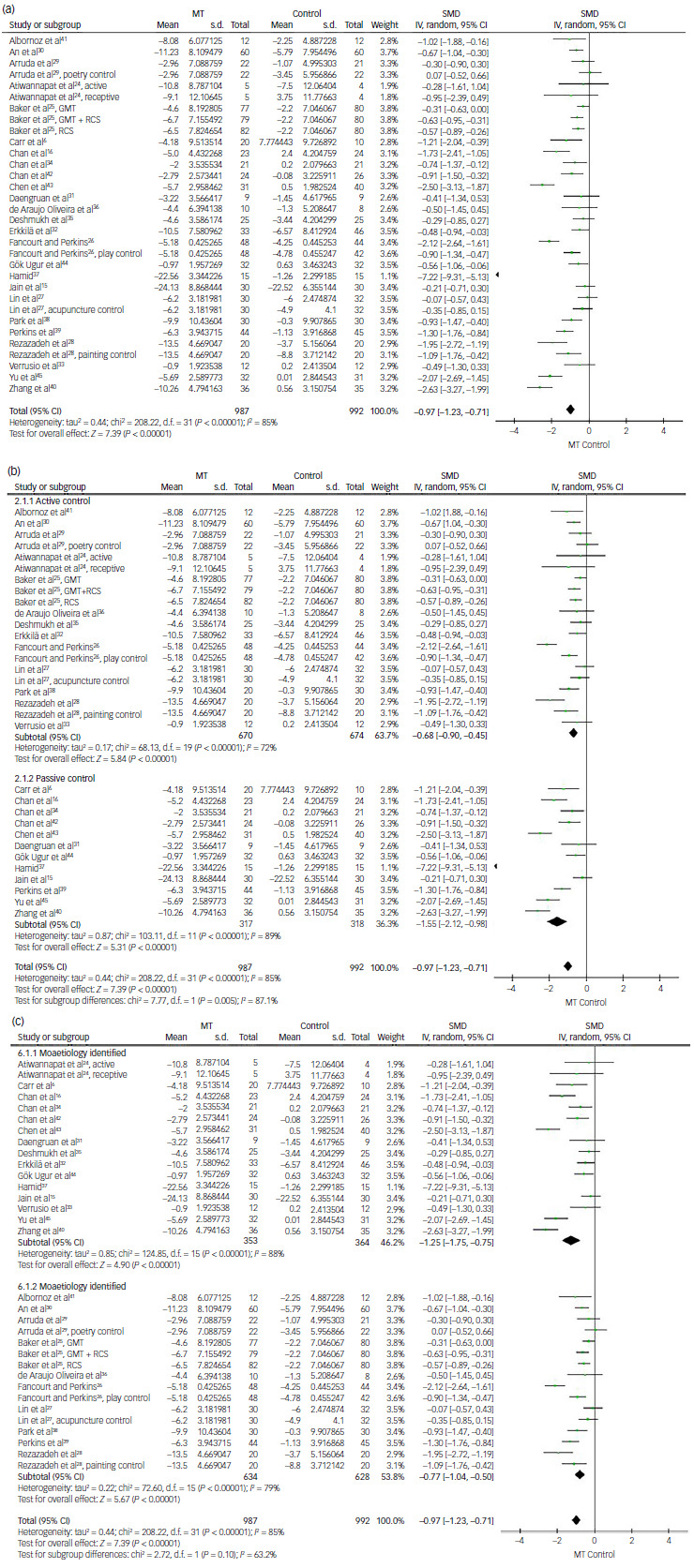
Comparison of depression scores: (a) music therapy (MT) versus control, (b) subgroup analysis for active and passive control, (c) subgroup analysis for not identified or identified aetiology or underlying diseases. IV, inverse variance; SMD, standardised mean difference; GMT, group music therapy; RCS, recreational choir singing.

For analysis of depression score, 32 comparisons from 25 studies provided continuous data of depression scores for the analysis of effect size using SMD. The overall mean difference was estimated to be –0.97 (95% CI: −1.23 to −0.71), suggesting that music therapy showed greater reduction in depression score with statistical significance (*P* < 0.01). Almost all included trials, except one, demonstrated favourable outcomes for music therapy in a forest plot. However, individual mean differences varied across studies. For example, Zhang et al reported a mean difference of −2.63 whereas Arruda et al reported a value of 0.07, slightly favouring the control group.^29,40^ A high degree of heterogeneity was observed (*I* ^2^ = 85%, *P* < 0.01) (Fig. 3a).

Music therapy demonstrated significantly better effectiveness in controlling depression compared with both active and passive controls, with statistical significance (*P* < 0.01). The overall mean differences were −0.68 (95% CI: −0.90 to −0.45) and −1.55 (95% CI: −2.12 to −0.98) for active and passive controls, respectively (Fig. 3b). Similarly, the beneficial effect of music therapy remained statistically significant regardless of whether the aetiology or underlying diseases were identified (*P* < 0.01). The overall mean differences were −0.97 (95% CI: −1.23 to −0.71) in studies with identified underlying conditions and −1.25 (95% CI: −1.75 to −0.75) in those without specified aetiology (Fig. 3c).

#### Music therapy for depression according to music therapy type (active music therapy, receptive music therapy and music medicine)

**Fig. 4:**
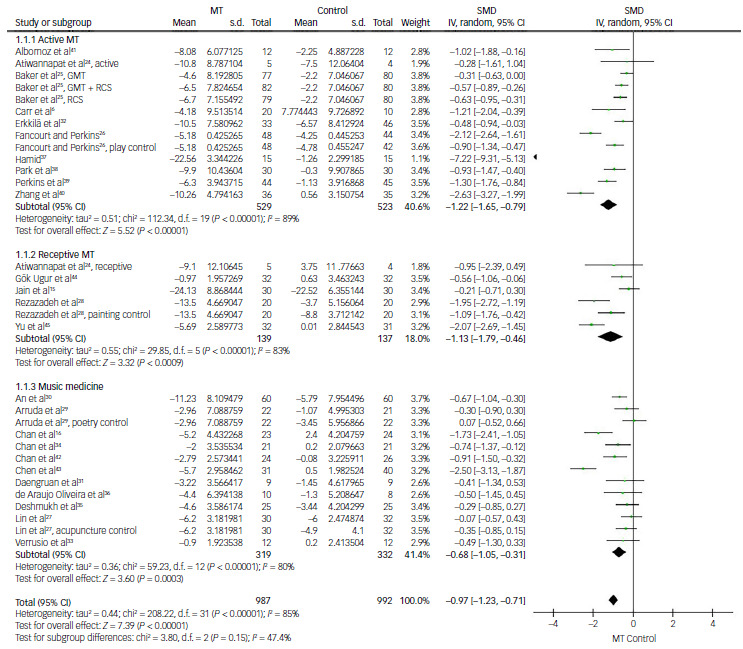
Comparison of depression scores: active music therapy (MT), receptive MT and music medicine versus control. Active MT, active music therapy; receptive MT, receptive music therapy; IV, inverse variance; GMT, group music therapy; RCS, recreational choir singing; SMD, standardised mean difference.

The first subgroup analysis was conducted following dividision of the studies into three subgroups based on music therapy type. In the active music therapy subgroup, 13 comparisons from 10 studies were available in the analysis of effect size using the SMD of depression score improvement.^ 6,24–26,32,37–41^ The overall SMD was estimated to be −1.22 (95% CI: −1.65 to −0.79), indicating a statistically significant improvement favouring active music therapy (*P* < 0.01). In the receptive music therapy subgroup, which included 6 comparisons from 5 studies,^15,24,28,44,45^ SMD was estimated to be −1.13 (95% CI: −1.79 to −0.46), also suggesting a statistically significant improvement compared with controls (*P* < 0.01). For music medicine, 13 comparisons from 11 studies provided relevant data,^16,27,29–31,33–36,42,43^ with an overall SMD of −0.97 (95% CI: −1.23 to −0.71), indicating a significant reduction in depression score (*P* < 0.01). Although all three music therapy types produced statistically significant improvements, active music therapy appeared to have yielded the largest mean difference, followed by receptive music therapy and music medicine, respectively. Notably, within the music medicine subgroup, three studies showed distinctly larger mean differences than the othes.^16,34,42^ These studies used rest as the control group, whereas other studies usually used active controls such as conventional treatment. A high degree of heterogeneity was observed across all three subgroups (*I* ^2^ = 89, 83 and 80%, respectively; *P* < 0.01) (Fig. 4).

#### Music therapy for depression according to music therapy delivery method (group- or individual-based music therapy)

**Fig. 5:**
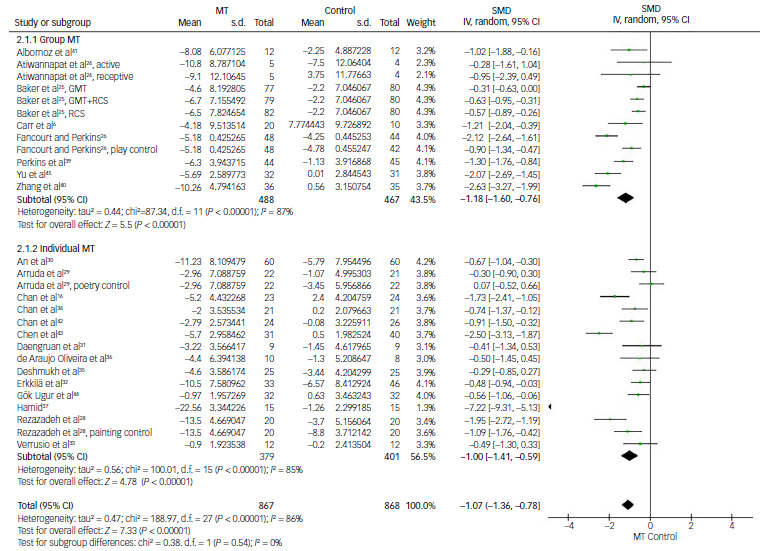
Comparison of depression scores: group music therapy (MT) and individual MT versus control. Group MT, group-based music therapy; individual MT, individual-based music therapy; IV, inverse variance; GMT, group music therapy; RCS, recreational choir singing; SMD, standardised mean difference.

The second subgroup analysis was conducted following dividision of the studies into two subscales based on whether music therapy was conducted by group or individually. In the group-based music therapy subgroup, data were obtained for 12 comparisons from 8 studies.^ 6,24–26,39–41,45^ The SMD was measured as −1.18 (95% CI: −1.60 to −0.76), suggesting that the music therapy group had a more significant reduction in depression score than the control group (*P* < 0.01). For individual-based music therapy, 16 comparisons from 14 studies yielded an SMD of −1.0 (95% CI: −1.41 to −0.59),^16,28–37,42–44^ showing a significant reduction in depression score compared with the control group (*P* < 0.01). Although both groups appear to be effective, the mean difference seemed to be relatively larger in the group-based music therapy subgroup. Notably, studies by Fancourt and Perkins, Yu et al and Zhang et al in the group-based music therapy subgroup reported larger effect sizes than others.^26,40,45^ A high degree of heterogeneity was observed in both subgroups (*I* ^2^ = 87 and 85%; *P* < 0.01) (Fig. 5).

#### Music therapy for depression according to staff profession (certified music therapist or non-music therapist)

**Fig. 6:**
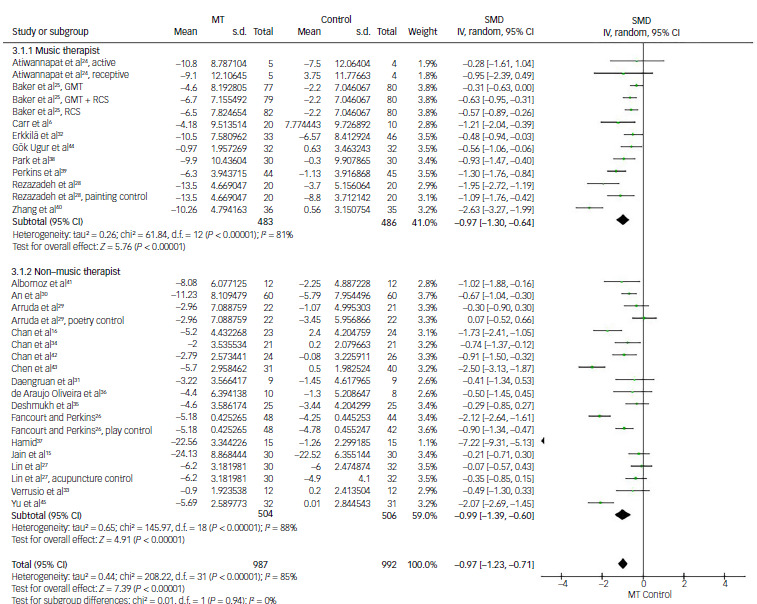
Comparison of depression scores: music therapist and non-music therapist versus control. MT, music therapy; IV, inverse variance; GMT, group music therapy; RCS, recreational choir singing; SMD, standardised mean difference.

The third subgroup analysis was conducted following division of the studies into two subgroups depending on whether music therapy was guided by a certified music therapist or a non-music therapist. In the music therapist subgroup, 13 comparisons from 9 studies were available for the analysis of effect size using the SMD of depression score improvement.^ 6,24,25,28,32,38–40,44^ Three comparisons from Baker’s study showed a smaller mean difference favouring music therapy over other comparisons. In the non-music therapist subgroup, 19 comparisons from 16 studies provided data for the analysis.^15,16,26,27,29–31,33–37,41–43,45^ Both groups showed statistically significant reductions in depression scores compared with control, with SMDs of −0.97 (95% CI: −1.30 to −0.64; *P* < 0.01) and −0.99 (95% CI: −1.39 to −0.60; *P* < 0.01), respectively. The degree of heterogeneity for the groups was 81 and 85%, respectively (*P* < 0.01) (Fig. 6).

#### Music therapy versus art therapies for depression

Music therapy was compared with other art therapies, including painting, poetry and creative play, among controls to investigate whether music therapy has a greater effectiveness in improving depression scores than other art therapy types. This analysis, made by 6 comparisons from 4 studies, revealed a SMD of −0.56, favouring music therapy with statistical significance (95% CI: −0.82 to −0.31; *P* < 0.01).^25,26,28,29^

**Fig. 7:**
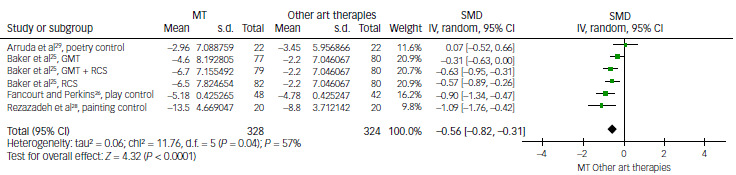
Comparison of depression scores: music therapy (MT) versus other art therapies. GMT, group music therapy; IV, inverse variance; RCS, recreational choir singing; SMD, standardised mean difference.

Fancourt and Perkins’ study, involving women with postnatal depression, demonstrated that the music therapy group showed significant improvement in depressive symptoms, whereas neither the control nor the creative play group exhibited such improvement.^26^ Rezazadeh et al’s study of children aged 6–12 years with burn injuries found that the music therapy group achieved significantly greater improvement in depression than the painting group.^28^ Arruda et al reported that the music therapy and poetry groups produced significant improvement in depressive symptoms, with no significant difference in adult patients with cancer pain.^28^ In Baker’s study of patients with dementia, the choir singing group showed significantly better results than the control group (which included activities such as group games, entertainment, art classes and gardening), while the music therapy group did not yield better results.^25^ The level of heterogeneity was measured as high (*I* ^2^ = 57%, *P* = 0.04) (Fig. 7).

#### Music therapy for QOL

**Fig. 8:**
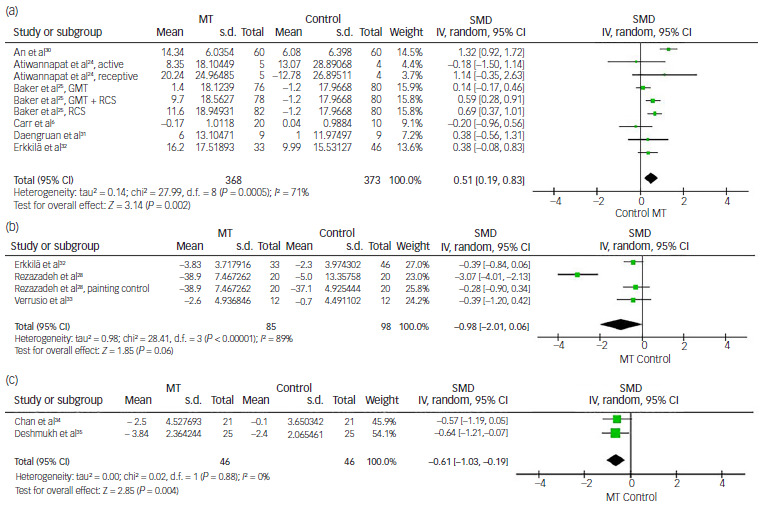
Comparison of (a) quality of life scores, (b) anxiety scores and (c) sleep quality scores: music therapy (MT) versus control. IV, inverse variance; GMT, group music therapy; RCS, recreational choir singing; SMD, standardised mean difference.

Nine comparisons from six studies provided continuous data regarding increased QOL scores for the analysis of effect size, using SMD.^ 6,24,25,30–32^ Overall SMD was estimated to be 0.51 (95% CI: 0.19 to 0.83), suggesting that QOL improvement was significantly better in the music therapy group than in the control group (*P* < 0.01). A high degree of heterogeneity was observed (*I* ^2^ = 71%, *P* < 0.01) (Fig. 8a).

#### Music therapy for anxiety

Four comparisons from three studies provided continuous data for the measurement of effect size on anxiety score reduction, using SMD.^28,32,33^ This suggested that music therapy exhibited greater reduction in anxiety score than the control group, with an effect size of −0.98 (95% CI: −2.01 to 0.06) but without statistical significance (*P* = 0.06). A significantly high level of heterogeneity was observed (*I* ^2^ = 89%, *P* < 0.01) (Fig. 8b).

#### Music therapy for sleep quality

Two studies were included in the analysis of effect size for sleep score improvement, using SMD.^34,35^ The overall SMD was estimated to be −0.61 (95% CI: −1.03 to −0.19), which favoured music therapy with statistical significance (*P* < 0.01). The level of heterogeneity was low (*I* ^2^ = 0%, *P* = 0.88) (Fig. 8c).

#### Level of evidence

The ROB was considered high (problematic or serious), as previously mentioned. Directness was not considered problematic because all the included studies directly compared music therapy with other controls. Funnel plot analyses were conducted in the category of music therapy for depression, and in the subgroup analysis of group-based music therapy, individual-based music therapy, active music therapy, music medicine and music therapist and non-music therapist for depression, because more than ten studies were included for each category. Although all funnel plots demonstrated asymmetry, this could not be completely attributed to publication bias due to the high level of heterogeneity across the selected studies. Consistency was considered problematic owing to clinical heterogeneity across the selected studies and statistical heterogeneity, as demonstrated by *I* ^2^ values, in all the meta-analysis categories. In addition, the degree of precision was regarded as problematic due to the small number of patients in the selected studies. Consequently, the evidence level as determined using the GRADE system was evaluated as being very low (Table 4).

**Table 4:** The Grading of Recommendations Assessment, Development and Evaluation system

Certainty assessment							No. of patients		Effect		Importance
No. of studies	Study design	Risk of bias	Inconsistency	Indirectness	Imprecision	Other considerations	Music therapy	Control	Relative (95% CI)	Absolute (95% CI)	
32	Randomised trials	Serious^a^	Serious^b^	Not serious^c^	Serious^d^	None	987	992	–	SMD 0.97 lower (1.23 lower to 0.71 lower)	Important

SMD, standardised mean difference.

a Overall, 97 (53.3%) of 182 domains were rated as low risk.

b Clinical and statistical heterogeneity.

c All the included studies directly compared music therapy with other controls.

d Small number of patients in the selected studies.

## Discussion

Depression is associated with significant structural alterations and functional disruptions in the cortical and limbic circuits that regulate mood and emotion. Elevated plasma cortisol levels are relevant to the suppression of neural plasticity, causing the enhancement of negative coping mechanisms for emotional problems. Furthermore, deficiency of neurotransmitters such as serotonin, and abnormal functioning of their receptors, are implicated in the pathophysiology of depression.^18^ A study investigating the effectiveness of music therapy in controlling depression in children with attention-deficit hyperactivity disorder observed improvement in the depression scale, as well as serotonin activation and cortisol reduction, in the music therapy group.^38^

Auditory stimulation by music pitch and tones can modulate emotional disturbances by influencing the limbic system, which plays a key role in memory, learning, motivation and emotional states.^16,17^ Aside from the limbic system, music enhances connectivity between the auditory cortex and other areas related to emotional control. In addition, it facilitates motor coordination, cognitive functions such as memory, attention span and behavioural augmentation, which contribute to improved emotional and neurological status.^46^ Music possesses an inherent ability to alleviate depression and other emotional distress, irrespective of underlying disease conditions, by modulating neurotransmitters or the interconnection between various brain regions.^47^ Our findings also support the effectiveness of music therapy, regardless of delivery method or associated disease entities. In addition, the therapeutic benefit of music therapy was not observed in comparison with only passive controls, such as just waiting or rest, but also in comparison with active controls, including psychotherapy and pharmacology. However, many of the included studies established diverse controls and their combinational use – for example, pharmacotherapy and psychotherapy, which made it difficult to ensure that music therapy was superior to all types of control treatments. Meta-analyses demonstrated that music therapy showed statistically significant improvement in depression compared with various controls that were not subdivided,^1,48^ while another analysis was undecided as to whether music therapy outperformed psychological therapies in controlling depression.^12^

Active music therapy, involving direct and active participation in producing and playing music, helped to establish interpersonal links between patients and therapists or other patient groups. In addition, active music therapy supports the development of problem-solving skills and enhances a feeling of self-worth or achievement.^40^ The creation and expression of musical sounds by patients helped to alleviate emotional problems, and to improve immunity, physical performance and QOL.^49–52^ These properties of active music therapy are presumed to underlie its effectiveness in alleviating depression compared with receptive music therapy or music medicine. Active music therapy was reported to be more efficient than music listening for children with autistic spectrum disorders.^53^ Contrarily, a meta-analysis indicated that receptive music therapy was better than active music therapy in controlling depression. This finding was attributed to the tendency of individuals with depression to adopt a passive attitude and exhibit reduced motivation for active engagement. ^48^ Therefore, the choice between active and passive music therapy may depend on an individual’s characteristics, including the severity and nature of their depressive symptoms.

The relative advantage of group- or individual-based therapy remains a subject of debate. One study showing results contrary to our findings stressed the advantage of an individual-based approach, arguing that it allows for more tailored interventions that address specific subject needs. That study highlighted that group-based therapy, which centres on human interactions, may not effectively meet the needs of those with depression who have a tendency for weak social interactions and increased social avoidance.^48^ However, social avoidance or insufficiency was also one of the key factors in managing depressive symptoms. In this context, group-based therapy could be more clinically beneficial or effective than individual-based therapy. Many articles have stated that group-based therapy promotes social bonding by improving interpersonal relationships, placing the individual in contact with their musical and psychological resources and allowing acceptance of others in socialised activities, which, in turn, provides opportunities to build inner resources of coping, resilience and hope.^ 6,40,54,55^

Contrary to expectation, music therapy guided by certified professional therapists failed to show significant benefits compared with that guided by non-professional staff. However, despite this finding, given that group-based or active music therapy, which is more dependent on specialised therapists, appeared to be better than individual-based or receptive music therapy, respectively, there was a probability that a professional music therapist might be more advantageous in regard to more effective music therapy. The lack of statistical significance regarding the professional background of the intervention provider in this review can be attributed to the characteristics of the study population. Four comparisons from two studies performed by a music therapist showed a smaller mean difference favouring music therapy, which might have contributed to statistical insignificance.^25,44^ In both studies, the participants were elderly patients with dementia or in a nursing home. It is plausible that advanced age and cognitive dysfunction had a more pronounced influence on the outcome than the professional qualifications of the staff.

Music therapy exhibited greater clinical effectiveness in reducing depressive symptoms than other active art therapies. However, inconsistent results were observed across the included studies, possibly because the art therapies used as control and the characteristics of participants in the selected studies were highly variable. To the best of our knowledge, no study has clearly defined whether music therapy is better than other art therapies, or provided strong, supportive evidence for such a claim. Although it is being cautious to assert that music therapy was better than other art therapies for depression based solely on the current study, music might be considered a better option than other arts in managing emotional problems. This is supported by the evidence that auditory stimuli from musical sound activate the limbic system, which is closely related to emotional control, thus helping to relieve psychological stress and depression.^56^

Four studies included in this analysis showed a trend in which the superiority of music therapy over other art therapies was more prominent in young patients and children, but diminished in patients of older age or in those with more serious medical problems, such as cancer and dementia. Based on this tendency, music therapy may be more effective in younger patients with less severe disease and intact cognitive function, who are able to accept and understand music more efficiently and communicate with both peers and therapists.

Music therapy promoted interpersonal communication and created a harmonious environment, which may contribute to the amelioration of anxiety symptoms. Furthermore, music therapy encouraged patients to concentrate on the rhythm of the music, thereby distracting them from their underlying disease and its associated problems, including the emotional distress experienced during treatment. Music also played a role in both relaxing tightened muscles and hyperarousal status, which induced a positive change in the subject’s mental state and improved sleep condition.^57,58^ Ultimately, music therapy resulted in the improvement of QOL by reducing emotional problems, enhancing sleep quality and providing distraction from underlying disease and associated problems.^59^

This study has several limitations. First, the quality of some included studies is a concern, mainly because of the unclear allocation sequence concealment, lack of blinding of participants and personnel and lack of blinding of outcome assessment. Second, the degree of statistical and clinical heterogeneity was high in almost all analyses, which reduced the statistical power of the meta-analysis. Third, the number of participants was small in a considerable number of studies, affecting the confidence intervals of some categories and therefore limiting the precision and accuracy of the results. These limitations ultimately lowered the evidence level and strength of recommendation.

Fourth, subgroup analysis according to music therapy intensity, considered an important point of music therapy, was not performed. One previous meta-analysis conducted subgroup analysis according to high- and low-intensity groups (60 min/week or less versus >60 min/week).^14^ However, that study also did not account for the number of sessions per week or the total number of sessions, which are important factors in determining treatment intensity. In our review, we were unable to obtain sufficient data regarding music therapy protocols across the selected studies.

Fifth, the included studies involved various underlying aetiologies, which could have introduced bias when concluding the effect of music therapy on depression. We conducted subgroup analysis only, by dividing the studies into those with identified or unidentified aetiologies. This study primarily focused on the therapeutic effects of music therapy based on various methodological subcategories, including subtype, delivery methods and professionality, in relation to reducing depression-related clinical symptoms. Music therapy was not considered a treatment for underlying diseases, but rather a method to manage emotional symptoms.^1,46,47^ In this context, we did not choose a specific diagnosis or aetiology. Furthermore, many articles included in this study did not limit participants by a specific diagnosis. Instead, they applied broad eligibility criteria, such as those diagnosed with depression based on a self-rated questionnaire or a clinician-rated assessment method, regardless of the underlying morbidity.^ 6,15,16,23,24,31–35,37,40,42–45^ As a result, sufficient data for subgroup analysis by aetiology or underlying disease were not available. Similarly, previous systematic reviews with meta-analyses published up to that point in time, including the Cochrane review, also focused on depression itself, irrespective of aetiology.^10,12,48,54^ A Cochrane review stated that they included participants with comorbidities such as anxiety disorder, alcohol abuse, personality disorder, dementia, autism, schizophrenia, psychosis and somatoform disorder, recognising that depression often co-occurs with other diagnoses.^12^ However, when sufficient data become available, conducting a meta-analysis based on different underlying diseases would provide valuable insights.

Overall, the synthesised results of the selected studies and meta-analysis indicate that music therapy might be superior, or at least not inferior to, various controls, including pharmacotherapy, psychotherapy and even other art therapies, in the treatment of depression and related clinical aspects such as QOL, anxiety and sleep conditions. This advantage of music therapy could be achieved regardless of whether it is employed as active music therapy, receptive music therapy or music medicine; whether a professional therapist guided it; and whether it was performed in a group or individually. Music therapy can be applied to individuals without requiring high-cost, trained staff members or skillful techniques. Most people widely enjoy music, and it can therefore be used in those with depression without hesitation. In addition, music is not associated with serious adverse effects, offering reassurance to people who may be anxious about the potential side effects of pharmacotherapy.

In conclusion, considering that the meta-analysis of relevant studies demonstrated statistically significant superiority of music therapy over controls, along with the inherent characteristics of music – being popularly enjoyable, easily accessible, low in cost and free of serious side effects – music therapy could be recommended with weak strength for individuals with depression to control depressive symptoms and anxiety, and to improve QOL and sleep quality, despite the low level of evidence. Future studies analysing the effects of music therapy according to more specific underlying aetiologies or disease entities, once further RCTs have been accumulated, would provide more meaningful clinical implications.

## Supporting information

Lee et al. supplementary material Lee et al. supplementary material

## Data Availability

Extracted data are available on request from the corresponding author.

## Supplementary material

The supplementary material is available online at https://doi.org/10.1192/bjo.2025.10822
