# Supplementary material for: Music therapy for patients with depression: systematic review and meta-analysis of randomised controlled trials
Source: BJPsych Open. 2025 Sep 9;11(5):e201. doi: 10.1192/bjo.2025.10822 (PMC12451534; doi:10.1192/bjo.2025.10822)
Supplement: Lee et al. supplementary material [file S2056472425108223sup001.docx]

|  | **no.** |  |
| --- | --- | --- |
| **PubMed** | #1 | "Depression"[Mesh] |
|  | #2 | "Depressive Symptoms"[TW] OR "Depressive Symptom"[TW] OR "Symptom, Depressive"[TW] OR "Emotional Depression"[TW] OR "Depression, Emotional"[TW] |
|  | #3 | "depression"[TW] |
|  | #4 | "Depressive disorder"[Mesh] |
|  | #5 | "Depressive Disorders"[TW] OR "Disorder, Depressive"[TW] OR "Disorders, Depressive"[TW] OR "Neurosis, Depressive"[TW] OR "Depressive Neuroses"[TW] OR "Depressive Neurosis"[TW] OR "Neuroses, Depressive"[TW] OR "Depression, Endogenous"[TW] OR "Depressions, Endogenous"[TW] OR "Endogenous Depression"[TW] OR "Endogenous Depressions"[TW] OR "Depressive Syndrome" [TW] OR "Depressive Syndromes" [TW] OR "Syndrome, Depressive"[TW] OR "Syndromes, Depressive"[TW] OR "Depression, Neurotic"[TW] OR "Neurotic Depression"[TW] OR "Neurotic Depressions"[TW] OR "Melancholia"[TW] OR "Melancholias"[TW] OR "Unipolar Depression"[TW] OR "Depression, Unipolar"[TW] OR "Depressions, Unipolar"[TW] OR "Unipolar Depressions"[TW] |
|  | #6 | "Depressive disorder"[TW] |
|  | #7 | "Dysthymic Disorder"[Mesh] |
|  | #8 | "Disorder, Dysthymic"[TW] OR " Dysthymic Disorders"[TW] OR"Dysthymia"[TW] OR "Persistent Depressive Disorder, Dysthymia"[TW] OR "Dysthymia and Chronic Depression"[TW] |
|  | #9 | "dysthymia"[TW] |
|  | #10 | "Bipolar Disorder"[Mesh] |
|  | #11 | "Bipolar Disorders"[TW] OR "Disorder, Bipolar"[TW] OR "Psychosis, Manic-Depressive"[TW] OR "Psychosis, Manic Depressive"[TW] OR "Psychoses, Manic-Depressive"[TW] OR "Psychoses, Manic Depressive"[TW] OR "Manic-Depressive Psychosis"[TW] OR "Manic Depressive Psychosis" [TW] OR "Bipolar Mood Disorder"[TW] OR "Bipolar Mood Disorders"[TW] OR "Disorder, Bipolar Mood"[TW] OR "Mood Disorder, Bipolar"[TW] OR "Affective Psychosis, Bipolar"[TW] OR "Bipolar Affective Psychosis"[TW] OR "Psychoses, Bipolar Affective"[TW] OR "Psychosis, Bipolar Affective"[TW] OR "Manic Depression"[TW] OR "Depression, Manic"[TW] OR "Depressions, Manic"[TW] OR "Depression, Bipolar"[TW] OR "Bipolar Depression" [TW] OR "Manic Disorder"[TW] OR "Disorder, Manic"[TW] OR "Manic Disorders"[TW] |
|  | #12 | "bipolar disorder"[TW] |
|  | #13 | "Cyclothymic Disorder"[Mesh] |
|  | #14 | "Cyclothymic Disorders"[TW] OR "Disorder, Cyclothymic"[TW] OR "Cyclothymic Personality"[TW] OR "Cyclothymic Personalities"[TW] OR "Personality, Cyclothymic"[TW] OR "Cyclothymia"[TW] |
|  | #15 | "cyclothymia"[TW] |
|  | #16 | "Mood disorders"[Mesh] |
|  | #17 | "Disorder, Mood" [TW] OR "Disorders, Mood" [TW] OR "Mood Disorder"[TW] OR "Affective Disorders"[TW] OR "Affective Disorder"[TW] OR "Disorder, Affective" [TW] OR "Disorders, affective"[TW] |
|  | #18 | "mood disorder"[TW] OR "affective disorder"[TW] |
|  | #19 | #1 OR #2 OR #3 OR #4 OR #5 OR #6 OR #7 OR #8 OR #9 OR #10 OR #11 OR #12 OR #13 OR #14 OR #15 OR #16 OR #17 OR #18 |
|  | #20 | "Music Therapy"[Mesh] |
|  | #21 | "Therapy, music"[TW] |
|  | #22 | "music"[TW] OR "music therapy"[TW] OR "music treatment"[TW] OR "vocal music"[TW] OR "instrumental music"[TW] |
|  | #23 | #20 OR #21 OR #22 |
|  | #24 | #19 AND #23 |
| **DB** | **no.** |  |
| **Embase** | #1 | 'depression'/exp |
|  | #2 | 'central depression':ti,ab,kw OR 'clinical depression':ti,ab,kw OR 'depressive disease':ti,ab,kw OR 'depressive disorder':ti,ab,kw OR 'depressive episode':ti,ab,kw OR 'depressive illness':ti,ab,kw OR 'depressive personality disorder':ti,ab,kw OR 'depressive state':ti,ab,kw OR 'depressive symptom':ti,ab,kw OR 'depressive syndrome':ti,ab,kw OR 'mental depression':ti,ab,kw OR 'parental depression':ti,ab,kw OR 'depression':ti,ab,kw |
|  | #3 | 'depression':ti,ab,kw,de |
|  | #4 | 'depressive disorder':ti,ab,kw,de |
|  | #5 | 'dysthymia'/exp |
|  | #6 | 'depression, neurotic':ti,ab,kw OR 'depressive neurosis':ti,ab,kw OR 'depressive reaction':ti,ab,kw OR 'dysthymic disorder':ti,ab,kw OR 'neurotic depression':ti,ab,kw OR 'dysthymia':ti,ab,kw |
|  | #7 | 'dysthymia':ti,ab,kw,de |
|  | #8 | 'bipolar disorder'/exp |
|  | #9 | 'bipolar affective disorder':ti,ab,kw OR 'bipolar and related disorders':ti,ab,kw OR 'bipolar illness':ti,ab,kw OR 'bipolar psychosis':ti,ab,kw OR 'depression, manic':ti,ab,kw OR 'manic depression':ti,ab,kw OR 'manic depression psychosis':ti,ab,kw OR 'manic depressive':ti,ab,kw OR 'manic depressive disease':ti,ab,kw OR 'manic depressive disorder':ti,ab,kw OR 'manic depressive illness':ti,ab,kw OR 'manic depressive psychosis':ti,ab,kw OR 'manic depressive reaction':ti,ab,kw OR 'manic depressive syndrome':ti,ab,kw OR 'maniodepressive psychosis':ti,ab,kw OR 'mano depressive syndrome':ti,ab,kw OR 'psychosis, manic depressive':ti,ab,kw OR 'bipolar disorder':ti,ab,kw |
|  | #10 | bipolar disorder':ti,ab,kw,de |
|  | #11 | 'cyclothymia'/exp |
|  | #12 | 'cyclophrenia':ti,ab,kw OR 'cyclothymic depression':ti,ab,kw OR 'cyclothymic disorder':ti,ab,kw OR 'cyclothymic personality':ti,ab,kw OR 'personality, cyclothymic':ti,ab,kw OR 'cyclothymia':ti,ab,kw |
|  | #13 | 'cyclothymia':ti,ab,kw,de |
|  | #14 | 'mood disorder'/exp |
|  | #15 | 'affective disorder':ti,ab,kw OR 'affective disorders':ti,ab,kw OR 'affective disturbance':ti,ab,kw OR 'affective illness':ti,ab,kw OR 'mood disorders':ti,ab,kw OR 'mood disturbance':ti,ab,kw OR 'mood disturbances':ti,ab,kw OR 'mood disorder':ti,ab,kw |
|  | #16 | 'mood disoder':ti,ab,kw,de OR 'affective disorder':ti,ab,kw,de |
|  | #17 | #1 OR #2 OR #3 OR #4 OR #5 OR #6 OR #7 OR #8 OR #9 OR #10 OR #11 OR #12 OR #13 OR #14 OR #15 OR #16 |
|  | #18 | 'music therapy'/exp |
|  | #19 | 'therapy, music':ti,ab,kw OR 'music therapy':ti,ab,kw |
|  | #20 | 'music':ti,ab,kw,de OR 'music therapy':ti,ab,kw,de OR 'music treatment':ti,ab,kw,de OR 'vocal music':ti,ab,kw,de OR 'instrumental music':ti,ab,kw,de |
|  | #21 | 'active music therapy'/exp |
|  | #22 | 'active group music therapy':ti,ab,kw OR 'active music therapy':ti,ab,kw |
|  | #23 | 'receptive music therapy'/exp |
|  | #24 | 'passive music therapy':ti,ab,kw OR 'passive musical therapy':ti,ab,kw OR 'receptive group music therapy':ti,ab,kw OR 'receptive musical therapy':ti,ab,kw OR 'receptive music therapy':ti,ab,kw |
|  | #25 | #18 OR #19 OR #20 OR #21 OR #22 OR #23 OR #24 |
|  | #26 | #17 AND #25 |
| **DB** | **no.** |  |
| **Cochrane Library** | #1 | MeSH descriptor: [Depression] explode all trees |
|  | #2 | Emotional Depression OR "Symptom, Depressive" OR "Depressive Symptom" OR "Depressive Symptoms" OR "Depression, Emotional" |
|  | #3 | "depression"[TW] |
|  | #4 | MeSH descriptor: [Depressive Disorder] explode all trees |
|  | #5 | Syndromes, Depressive OR "Depressive Syndrome" OR "Syndrome, Depressive" OR "Depressive Syndromes" OR "Endogenous Depression" OR "Depression, Endogenous" OR "Depressions, Endogenous" OR "Endogenous Depressions" OR "Neuroses, Depressive" OR "Neurosis, Depressive" OR "Disorder, Depressive" OR "Depressive Neurosis" OR "Disorders, Depressive" OR "Depressive Disorders" OR "Depressive Neuroses" OR "Neurotic Depressions" OR "Depressions, Neurotic" OR "Depression, Neurotic" OR "Neurotic Depression" OR "Melancholias" OR "Melancholia" OR "Unipolar Depression" OR "Depression, Unipolar" OR "Depressions, Unipolar" OR "Unipolar Depressions" |
|  | #6 | "Depressive disorder"[TW] |
|  | #7 | MeSH descriptor: [Dysthymic Disorder] explode all trees |
|  | #8 | Dysthymia and Chronic Depression OR "Neurotic Depression" OR "Persistent Depressive Disorder" OR "Dysthymia" OR "Disorder, Dysthymic" OR "Dysthymic Disorders" OR "Persistent Depressive Disorder, Dysthymia" |
|  | #9 | "dysthymia"[TW] |
|  | #10 | MeSH descriptor: [Bipolar Disorder] explode all trees |
|  | #11 | "Disorder, Bipolar Mood" OR "Psychosis, Manic Depressive" OR "Manic Depressive Psychosis" OR "Mood Disorder, Bipolar" OR "Psychoses, Bipolar Affective" OR "Psychosis, Bipolar Affective" OR "Affective Psychosis, Bipolar" OR "Psychosis, Manic-Depressive" OR "Bipolar Affective Psychosis" OR "Psychoses, Manic-Depressive" OR "Bipolar Mood Disorders" OR "Psychoses, Manic Depressive" OR "Bipolar Mood Disorder" OR "Manic-Depressive Psychosis" OR "Disorder, Manic" OR "Manic Disorder" OR "Manic Disorders" OR "Bipolar Disorders" OR "Disorder, Bipolar" OR "Bipolar Depression" OR "Depression, Bipolar" OR "Manic Depression" OR "Depressions, Manic" OR "Depression, Manic" |
|  | #12 | "bipolar disorder"[TW] |
|  | #13 | MeSH descriptor: [Mood Disorders] explode all trees |
|  | #14 | Mood Disorder OR "Affective Disorder" OR "Disorder, Mood" OR "Affective Disorders" OR "Disorders, Affective" OR "Disorder, Affective" OR "Disorders, Mood" |
|  | #15 | "mood disorder"[TW] OR "affective disorder"[TW] |
|  | #16 | MeSH descriptor: [Cyclothymic Disorder] explode all trees |
|  | #17 | "Cyclothymic Personalities" OR " Cyclothymic Disorders" OR "Cyclothymic Personality" OR "Personality, Cyclothymic" OR "Disorder, Cyclothymic" OR "Cyclothymia" |
|  | #18 | "cyclothymia"[TW] |
|  | #19 | AND(#1-#18) |
|  | #20 | MeSH descriptor: [Music Therapy] explode all trees |
|  | #21 | "Therapy, music" |
|  | #22 | "music"[TW] OR "music therapy"[TW] OR "music treatment"[TW] OR "vocal music"[TW] OR "instrumental music"[TW] |
|  | #23 | #20 OR #21 OR #22 |
|  | #24 | #19 AND #23 |
| **DB** | **no.** |  |
| **KMBASE** | #1 | depression AND music |
| **DB** | **no.** |  |
| **Psyinfo** | #1 | ("Depression" OR "Depressive Symptom" OR "Emotional Depression" OR "Depressive disorder" OR "dysthymia" OR " Dysthymic Disorder" OR "Bipolar Disorder" OR " Manic Depression" OR "Cyclothymia" OR "Cyclothymic Disorder" OR " Cyclothymic personality" OR "mood disorder" OR "affective disorder") AND ("music" OR "music therapy" OR "music treatment" OR "vocal music" OR "instrumental music") |
| **DB** | **no.** |  |
| **CINAHL** | #1 | (MH "Depression") OR (MH "Bipolar Disorder") OR (MH "Affective Disorders, Psychotic") |
|  | #2 | "Depression" OR "Depressive Symptom" OR "Emotional Depression" |
|  | #3 | "Depressive disorder" |
|  | #4 | (MM "Dysthymic Disorder") |
|  | #5 | "dysthymia" OR " Dysthymic Disorder" |
|  | #6 | "Bipolar Disorder" OR " Manic Depression" |
|  | #7 | (MM "Cyclothymic Disorder") |
|  | #8 | "Cyclothymia" OR "Cyclothymic Disorder" OR " Cyclothymic personality" |
|  | #9 | (MM "Affective Disorders") |
|  | #10 | "mood disorder" OR "affective disorder" |
|  | #11 | #1 OR #2 OR #3 OR #4 OR #5 OR #6 OR #7 OR #8 OR #9 OR #10 |
|  | #12 | (MM "Music Therapy") OR (MM "Music") OR (MM "Singing") |
|  | #13 | "music" OR "music therapy" OR "music treatment" OR "vocal music" OR "instrumental music" |
|  | #14 | #12 OR #13 |
|  | #15 | #11 AND #14 |
